# School-Age Child Routines: Adaptation and Validation Studies of the Portuguese Version of the Child Routines Questionnaire

**DOI:** 10.1007/s10862-023-10021-3

**Published:** 2023-01-26

**Authors:** Sofia O. Major, Marta P. Alves, Ana I. Cunha, Catarina F. Pereira, Sara Sytsma Jordan

**Affiliations:** 1 University of the Azores, Ponta Delgada, Portugal; 2 CINEICC, University of Coimbra, Coimbra, Portugal; 3 Research Center in Business Sciences (NECE-UBI), University of Beira Interior, Covilhã, Portugal; 4 CIDESD, University of Beira Interior, Covilhã, Portugal; 5 University of Beira Interior, Covilhã, Portugal; 6 School of Psychology, University of Southern Mississippi, Hattiesburg, MS US; 7 Faculdade de Ciências Sociais e Humanas, Universidade dos Açores, Campus de Ponta Delgada, Rua da Mãe de Deus, 9500-321 Ponta Delgada, Portugal

**Keywords:** Child routines, school-age, validation, CRQ

## Abstract

Child routines have been recognized as positive contributors to children’s development. However, in Portugal there is still a lack of instruments available to assess school-age child routines. The purpose of this study was to present the translation, adaptation, and validation studies of the Portuguese version of the Child Routines Questionnaire (CRQ), a parent self-report measure developed to assess school-age child routines. A total of 460 parents of children aged between 6 and 12 years-old participated in the study. Two studies were conducted to define the CRQ-PT factor structure. In Study 1 (*n* = 204 children from 6 to 12 years-old), findings from the exploratory factor analysis provided evidence for a four-factor structure (for 32 items), which explained 43.53% of the total variance. In Study 2 (*n* = 256 children from 6 to 9 years-old), results from confirmatory factor analysis showed good model fit indices (CFI = 0.84, RMSEA = 0.06). The total scale of the CRQ-PT (*α* = 0.89) and its subscales showed good internal consistency. Further evidence of construct validity was shown by weak to moderate correlations with measures of parental sense of competence and family mealtime routines. Relevant contributions of the study are underscored, namely the availability and usefulness of a reliable and valid assessment tool to evaluate the routines of Portuguese school-age children for clinical practice and research purposes.

## Introduction

Families organize their daily life around a set of routines, which allow their members to carry out daily activities in a structured and efficient way (Fiese et al., 2002). According to Howe (2002), family routines can be described as standardized interactions, which are episodic (their beginning and end are clearly defined), cyclic (with a regular period of recurrence), and occur with an internal regularity (interactions follow a similar pattern whenever the routines are performed). Family routines represent a protective factor for family well-being, as they provide a sense of stability, continuity, and security over time (Migliorini et al., 2011). For children, they represent one of the ideal opportunities to practice and develop their self-regulatory skills, as they carry out daily activities (Brody & Flor, 1997; Ren & Fan, 2019; Ren & Xu, 2019). Through the development of self-regulatory skills, routines also foster children’s social-emotional adjustment (e.g., initiative, self-control, appropriate social behaviors) (Ren & Xu, 2019). In turn, this will be reflected in adaptation to the school environment, as the child will be able to self-regulate, manage behaviors, and engage in learning activities with peers (Ferretti & Bub, 2017; Muñiz et al., 2014). Over the last few decades, some measures to assess routines have been developed, however, in Portugal there is a lack of instruments specifically developed to assess the routines of school-age children. The availability of a valid and reliable tool that can assist in the assessment of child routines is needed.

Child routines are observable and repetitive behaviors that occur in a predictable and orderly manner throughout a child’s day-to-day life. Child routines typically include both the child and an adult caregiver who may assist and/or supervise the child’s activities (Sytsma et al., 2001). Parents, through the implementation of consistent and predictable routines, provide their children with environmental stimuli that promote children’s compliance and ensure that they perform daily tasks, in line with parental expectations (Sytsma et al., 2001; Urcuioli 2005). Furthermore, a stable and peaceful family environment encourages the active participation of children in planning and carrying out daily activities, allowing them to become active agents in their educational process (Spagnola & Fiese, 2007).

School-age child routines essentially comprise tasks related to school (e.g., studying, doing homework), meals (e.g., helping with meal preparation, setting the table), household chores (e.g., helping to tidy up), bedtime (e.g., putting on pajamas), personal hygiene (e.g., taking a shower) and leisure activities (e.g., playing, watching TV, listening to music, playing videogames) (Sytsma et al., 2001; Weisner, 2002). A typical morning routine involves a standardized sequence of behaviors, which include washing the face, brushing teeth, getting dressed, making the bed, having breakfast, and going to school (Sytsma et al., 2001; Wittig, 2005). A bedtime routine can include taking a shower, putting on the pajamas, brushing teeth, saying goodnight to family members, reading a story, and going to bed (Kitsaras et al., 2018; Mindell & Williamson, 2018; Sytsma et al., 2001). According to McCloy et al., (2016), weekday routines tend to be more rigid and predominantly focused on self-care and productivity tasks, while weekend routines are more flexible, and prioritize leisure activities.

There is empirical evidence that interactions between parents and children during the practice of daily routines allow for greater regulation of the child’s behavior and improvement in parents’ perception of their parenting skills (Fiese et al., 2002; Sprunger et al., 1985). In this sense, parents’ involvement in their children’s daily lives allows everyday monitoring and control over children’s behavior, which, consequently, promotes the development of a sense of parental competence (Evans & Rodger, 2008; Fiese et al., 2002; Rania et al., 2018). Research focused on the relationship between family routines and parental competence suggests that a stable and affective environment promotes positive interactions between parents and children (Brody & Flor, 1997; Spagnola & Fiese, 2007). In turn, this leads to the adoption of positive parenting practices and increased levels of parental self-efficacy, satisfaction, and competence, which consequently results in the implementation of structured and consistent family routines (Ferretti & Bub, 2017; Jordanr, 2003).

### Assessment of Child Routines

The assessment of routines was initially focused on the routines of the whole family. One of the first instruments to be developed was the Family Routines Inventory (FRI; Jensen et al., 1983) which aimed to assess family cohesion, solidarity, organization, predictability, and satisfaction with family life. The FRI consists of 28 items, grouped into 12 domains or areas of family functioning (e.g., workday, weekends and leisure time, children’s routines, sleep time, meals, discipline routines) (Jensen et al., 1983). Later, the Family Rituals Questionnaire (FRQ; Fiese & Kline, 1993) was developed to assess family rituals and routines through family members’ self-report. It consists of 56 items, associated to seven contexts (e.g., dinner time, weekends, holidays) and eight dimensions (e.g., roles, presence/participation, symbolic meaning). However, these two assessment tools are mainly focused on the structure and predisposition of the family unit (FRI) and family rituals (FRQ), as such are relatively restricted and not suitable to specifically assess routines of individual children. This is important because child outcomes such as externalizing behavior problems and clinical applications for parenting typically focus on behavior of a specific child in the family and siblings may have quite different routines on the basis of age and interests.

Based on these limitations, and on the scarcity of specific assessment instruments to measure routines of a specific child, new instruments focused on children’s development and that offered greater clinical utility were created (Wittig, 2005). For instance, the Childhood Routines Inventory (CRI; Evans et al., 1997) was developed with 19 items to assess the ritualistic, repetitive, and compulsive behaviors patterns during early childhood. Still, this instrument was not intended to assess school-age daily routines (Stabler, 2012; Wittig, 2005).

As such, based on the need of a standardized instrument that systematically could assess school-age child routines, the Child Routines Questionnaire (CRQ; Sytsma et al., 2001) was developed in the United States (US). It is a parent-report instrument, which aims to assess routines of school-age children (6–12 years old) in the family. It consists of 39 items rated on a five-point Likert scale, from 0 “*Never*” to 4 “*Nearly always*”, that indicates the frequency with which the child engages in a certain kind of routine during the last month. The development of the CRQ comprised three validation studies. In the first study, “Item development”, a large set of items were defined as representative of children’s daily routines and then reduced by a group of experts to a final set of 80 items. The second study, “Selection of items”, aimed to reduce this set of items to build a questionnaire with good internal consistency. The third study, “Item reduction, reliability and validity”, consisted of further item reduction and evaluation of the psychometric properties of the CRQ. The data were submitted to a factor analysis, using the principal component method, with Varimax orthogonal rotation. Factor solutions with three, four and five factors were run, with items being excluded for factor loadings ​​under 0.40 (Sytsma et al., 2001). The analyses pointed to a final structure of four factors, for 36 items, explaining 44.2% of the total variance. The items are organized in four subscales: Daily Living Routines, with 11 items related to activities of daily living, such as morning routine, bedtime routine, meals and typical family social interaction; Household Responsibilities, which includes nine items related to personal responsibilities, household chores and hygiene; Discipline Routines, which includes 11 items in reference to rules, discipline methods and structured family activities; and Homework Routines, with five items associated with homework and adult supervision (Sytsma et al., 2001). Three low base rate items (10, 20, and 30, that were not included in the factor solution) compose the Defensive Responding scale, which was designed as a validity scale to identify pattern responding or a tendency to report an unrealistically high frequency of routines (Jordan, 2003).

Several studies were conducted to offer evidence to support the reliability and validity of the CRQ. For internal consistency, a Cronbach’s alpha coefficient of 0.90 was obtained for the Total scale, 0.81 for the Daily Living Routines, 0.83 for the Household Responsibilities, 0.82 for the Discipline Routines and 0.79 for the Homework Routines subscales. A test-retest reliability 2–4 weeks’ study was conducted, with correlation coefficients ranging between 0.75 and 0.85 for the four subscales and 0.86 for the Total scale. For validity evidence, moderate inter-correlations were found between the CRQ subscales (*r* = 0.28 and 0.52). Further construct validity evidence of the CRQ was demonstrated through moderate positive correlation between the CRQ Total scale and the FRI frequency scale (*r* = 0.54) and between 0.20 and 0.50 for the CRQ subscales, with the lowest correlation for the Homework Routines subscale and the strongest for the Daily Living Routines subscale. Moderate negative correlations were found between the CRQ Total scale and the Eyberg Child Behavior Inventory (ECBI; Eyberg & Ross, 1978) Intensity scale (*r* = − 0.35) and correlations with the CRQ subscales ranging from − 0.14 (Discipline Routines) to − 0.33 (Daily Living Routines) (Sytsma et al., 2001).

As far as we know, only one study has adapted the CRQ to another language, giving rise to an Icelandic version of the instrument (CRQ-IS; Halldórsdóttir & Óskarsdóttir, 2009). After performing four, five and six factor solutions (principal component analysis method, with Varimax orthogonal rotation), the authors found that the four-factor structure proved to be the most adequate for the CRQ-IS. Six out of the original items were eliminated and the remaining 33 items were organized into four subscales: Household Responsibilities (nine items), Family Interaction (eight items), Daily Living Routines (nine items), and Discipline Routines (seven items). Reliability studies evidenced a Cronbach’s alpha coefficient of 0.83 for the Total scale and values ​​between 0.67 and 0.81 for the four subscales (Halldórsdóttir & Óskarsdóttir, 2009). Also, the CRQ has been adapted to assess parental perceptions of child routines in other age groups and as a self-report measure. Namely, the Child Routines Questionnaire: Preschool (CRQ:P; Wittig, 2005) was developed to be used with preschool-age children (1–5 years) and is already adapted to Portuguese (Cunha et al., 2021) and Chinese (Ren & Fan, 2019); the Adolescent Routines Questionnaire: Parent and Self-Report (ARQ:P/S; Meyer, 2008) is composed by one version for parents and another for adolescents self-report (12–17 years old); and the Child Routines Questionnaire: Child Self Report (CRQ-CSR; Stabler, 2012) is a self-report tool for children aged 8–12 years-old.

Since its development, the CRQ has been recognized as a useful instrument to measure child routines and understand their impact on children’s growth and development (Henderson et al., 2011). For instance, it has been used to analyze the association between routines and parenting practices (Jordan, 2003), adherence to treatment in chronic diseases (Greening et al., 2007), externalizing and internalizing behavior problems in community children (Bridley & Jordan, 2012), to study routines in children with Attention Deficit Hyperactivity Disorder (ADHD; McRae et al., 2020; Pennick, 2013) or Autism Spectrum Disorder (ASD; Henderson et al., 2011; Stoppelbein et al., 2016), and to assess the effects of a parenting intervention program (Nymoen, 2014). More recently, an adapted version of the CRQ (19 items) was used to analyze whether daily routines during quarantine were potential protective factors in the association between COVID-19 cases in adolescents and post-quarantine depressive symptoms (Ren et al., 2021).

### The Current Study

In Portugal, there are two measures available to assess routines in the family context, namely the FRQ (Crespo et al., 2008) and the CRQ:P (Cunha et al., 2021). However, none is focused specifically on the assessment of routines of school-aged children. As such, the main purpose of this study was to translate, adapt and validate the CRQ (Sytsma et al., 2001) for Portuguese school-age children. More specifically, this study aimed to: (a) identify the factor structure for the Portuguese version (CRQ-PT) and to compare it with the original version (CRQ; Sytsma et al., 2001), (b) analyze the internal consistency and inter-correlations of all CRQ-PT scores, (c) confirm the factor structure previously obtained, and (d) provide further evidence of validity, by examining how CRQ-PT scores relate to measures of family routines and parental sense of competence. Since the CRQ-PT measures child routines in the family context, we expected that it would be positively associated with the dinner time subscale of the FRQ (Fiese & Kline, 1993), which has been used to assess mealtime routines in families with children (e.g., Friend et al., 2015; Jones et al., 2014). In line with research that suggests that routines in the family are associated with parental competence (e.g., Ferretti & Bub, 2017; Spagnola & Fiese, 2007; Jordan, 2003), we also expected that the CRQ-PT scores would positively correlate with perceived parenting efficacy and satisfaction, as evaluated by the Parenting Sense of Competence Scale (PSOC; Johnston & Mash, 1989).

## Method

### Participants

Four hundred and sixty parents, with at least one school-age child, participated in the study. Based on this sample, two studies were carried out. The first sample (Study 1) included 204 parents of children aged between 6 and 12 years old (58.8% boys; *M*age = 8.36 years; *SD* = 1.65). In most cases the child had siblings (73.5%). Most participants were mothers (82.8%) and married (82.4%). Participants reported their own as well as the other parent’s age and educational level. Mothers’ age ranged from 30 to 52 years (*M*age = 40.08 years; *SD* = 4.93) and fathers’ age from 22 to 61 years (*M*age = 43.43 years; *SD* = 6.30). Parents’ educational level was determined based on four levels of education: up to 6 years, 9 years, 12 years (high school), and more than 12 years (licence/bachelor, masters, and doctoral degrees). More than half of mothers (53.4%) and 35.8% of fathers were college graduated and the majority were employed (90.7% of mothers and 89.2% of fathers).

The second sample (Study 2) was comprised by 256 parents of children between 6 and 9 years old (54.2% girls; *M*age = 7.62 years; *SD* = 1.06), mostly with siblings (71.5%). Most participants were mothers (84.4%) and married (80.2%). Mothers’ mean age was 38.55 years (*SD* = 4.73; range 26–51 years) and father’s mean age was 41.07 years (*SD* = 5.95; range 26–69 years). About 43% of mothers and 25% of fathers were college graduated. Most parents were employed (83.9% of mothers and 96.4% of fathers). Sociodemographic characteristics of both samples are presented in Table 1.

**Table 1:** Sociodemographic characteristics of the samples

	Study 1		Study 2	
Characteristic	*N*	%	*N*	%
Child’s gender^a^				
Male	120	58.8	116	45.8
Female	84	41.2	137	54.2
Child’s age				
6 years	35	17.2	49	19.1
7 years	35	17.2	62	24.2
8 years	31	15.2	82	32.0
9 years	58	28.4	63	24.6
10 years	22	10.8	-	-
11 years	16	7.8	-	-
12 years	7	3.4	-	-
Mother’s education^a^				
Up to 6 years	7	3.4	18	7.0
9 years	36	17.6	54	21.1
12 years	51	25.0	73	28.5
> 12 years	109	53.4	109	42.6
Father’s education^b^				
Up to 6 years	16	7.9	29	11.4
9 years	50	24.5	66	25.8
12 years	56	27.7	84	32.8
> 12 years	73	35.8	64	25.0
Child siblings				
Yes	150	73.5	183	71.5
No	54	26.5	73	28.5

^a^Missing data from three participants, ^b^Missing data from 22 participants

### Procedure

This study is part of a larger research project about family and children’s health and was approved by the Ethics Committee of the University of Beira Interior (UBI). Data were collected both in school contexts and through informal networks. In the first case, the principals of selected schools were contacted to obtain permission for conducting the study. Research protocols were sent home with the children, with an informative letter about the study, the consent form, and parent-report questionnaires. Parents were asked to return the questionnaires in a sealed envelope to the child’s teacher one week later. In the second case, participants were recruited in person, with the assistance of undergraduate students attaining a master’s degree in psychology in the UBI. The inclusion criterion for Study 1 was to be a parent of a child between ages of 6 and 12 years old (same age range in the CRQ original study). For Study 2, the inclusion criterion was restricted to parents of children aged 6 to 9 years-old, considering that the parental sense of competence measure used in the present study was developed for preschoolers and children under 9 years-old.

### Instruments

#### Sociodemographic Questionnaire

Designed for this study, it was used to collect descriptive demographic information, including parents and child’s age and gender, child’s siblings, parents’ education, marital and professional status.

#### Child Routines Questionnaire (CRQ)

Developed by Sytsma et al. (2001), it was used to measure routines of school-aged children routines. Permission to adapt the instrument to Portuguese was requested and obtained from the authors of the original version. Following general guidelines for the translation process (Gjersing et al., 2010), the items of the CRQ were translated into Portuguese by two researchers from the team independently, and then revised by a third one to create a consensual version. This version was translated back by a fluently bilingual-speaking person and then the translation was compared with the original English version. Subsequently, a pilot study with five mothers of school-aged children was conducted, to certify the comprehensibility of the instructions, items, and response scale. Minor adjustments were made to ensure the accuracy of the final Portuguese version of the CRQ. Participants were asked to rate the frequency of the 39 listed routines on a 5-point Likert scale, ranging from 0 (“*Never*”) to 4 (“*Nearly always*”). Higher scores on the scale indicate the perception of more frequent routines.

#### Family Rituals Questionnaire (FRQ)

Family routines were assessed using the dinner time subscale of the FRQ (Fiese & Kline, 1993; Portuguese version: Crespo et al., 2008). The subscale is comprised of five items presented in a forced-choice format. First, participants indicate which description best represents their family (e.g., “Some families regularly eat dinner together” and “Other families rarely eat dinner together”) and then if that description is “Really true” or “Sort of true”. The four possible combinations of answers are scored on a 4-point Likert scale and higher scores indicate greater perception of routinization related to family meals. In the present study, Cronbach’s alpha reliability coefficient was 0.60.

#### Parenting Sense of Competence Scale (PSOC)

The PSOC (Johnston & Mash, 1989) is a 17-item self-report scale developed to assess perceived parenting competence, in parents of children aged 4 to 9 years old. Parents completed the Portuguese version (Seabra-Santos et al., 2015), composed by 16 items distributed across the original bifactor structure: Satisfaction (nine items) and Efficacy (seven items). Items are rated on a 5-point Likert scale (from “*Totally agree*” to “*Totally disagree*”). A total score and a score for each of the two dimensions can be calculated. Higher scores on the scales indicate a greater sense of parenting competence. In the present study, Cronbach’s alpha reliability coefficient was 0.78 for the Total scale.

### Analysis Plan

Statistical analyses were conducted using SPSS software (IBM SPSS Statistics 25 and IBM SPSS Amos version 25). Normality and missing values were checked for all the measures, considering each of the samples separately, and the cases which had at least 10% of the missing responses in one of the scales were eliminated (Bennett, 2001; Bryman & Cramer, 2004).

In Study 1 (*n* = 204), an exploratory factor analysis (EFA) was conducted using principal component analysis (PCA) and Varimax orthogonal rotation. In Study 2, the factor solution obtained was tested in another sample (*n* = 256) through a confirmatory factor analysis (CFA), using maximum likelihood (ML) estimation. Different indexes were used to assess model fit: Chi-Square (*χ*^*2*^ and *χ*^*2*^*/df*), Comparative Fit Index (CFI), Goodness-of-Fit Index (GFI) and Root Mean Square Error of Approximation (RMSEA, 90% CI) (Jackson et al., 2009). Based on suggested cut-off values, good model fit is associated with a small and significant *χ*^*2*^, values around 0.90 for CFI, and GFI and RMSEA below 0.10 (Byrne, 2010). Internal consistency was measured for all the scales and subscales with Cronbach’s alpha coefficients, considering values from 0.70 to 0.80 as respectable/good and values from 0.80 to 0.90 as very good (DeVellis, 2016). Finally, Pearson’s correlation was used to calculate associations between the CRQ-PT subscales and the Total score and between the CRQ-PT, FRQ and PSOC scores.

## Results

### Exploratory Factor Analysis

A series of exploratory factor analyses (Study 1) was conducted to achieve a model that would adequately fit our data. Only 36 out of the 39 original items were considered for analysis, since items 10, 20, and 30 had been excluded from the factor structure of the original version of the CRQ (Defensive Responding scale; Jordan, 2003). In addition, in this study, item 33 (“prayers before meals”) was eliminated, because it showed a very low mean frequency (*M* = 0.47; *SD* = 0.86) and it had also been removed in the CRQ-IS (Halldórsdóttir & Óskarsdóttir, 2009). Thus, a total of 35 items from the CRQ-PT were submitted to an EFA (using PCA and Varimax orthogonal rotation). These methodological decisions were consistent with the procedures used to validate the original version of the CRQ, as well as the CRQ-IS.

Preliminary analysis for suitability of this data set for factor analysis were conducted. The Kaiser-Mayer-Olkin (KMO) was 0.85 and the Bartlett test of sphericity reached statistical significance, *χ*^*2*^(595) = 2295.24, *p* < .001. The first EFA was calculated without any rotation or restriction of factors. To determine the number of components to be retained, the Kaiser-Guttman criterion, which considers the retention of factors with eigenvalue > 1 (Shrestha, 2021), was used. The analysis of the initial solution pointed to the extraction of nine components that presented eigenvalues greater than 1, explaining 59.56% of the total variance, but the first three components were responsible for more than half of the variance. Due to the excessive number of components, a second criterion was applied, the Cattell Scree Test (Cattell, 1966), suggesting the extraction of factors in a number between three and five. Thus, three EFA were run by restraining the extraction to three, four and five underlying components, respectively (an identical procedure used in the CRQ original version). To determine to which component an item should be assigned, the highest loadings were considered. Despite the different threshold loading values that can be found in the literature, for example 0.40 (Costello & Osborne, 2005) or 0.32 (Tabachnick & Fidell, 2007), in the present study, all the items that loaded at 0.38 or above on at least one component were retained, as it proved to be the most parsimonious option. Thus, after the elimination of three items (2, 24 and 32) with factor loadings lower than 0.38, the factor solution with four components showed the clearest structure and best represented the data set, both statistically and theoretically. Moreover, this solution also turned out to be congruent with the four-factor structure of the original version of the CRQ and the CRQ-IS. The factor loadings, communalities, explained variance, and descriptive statistics are displayed in Table 2.

**Table 2:** Descriptive statistics, factor loadings, communalities, and explained variance: CRQ-PT

CRQ-PT Items My child…			Components				
	*M*	*SD*	1	2	3	4	*h* ^*2*^
22. Helps clean up after meals	2.39	1.04	**0.77**	0.09	0.06	0.06	0.61
8. Cleans up food mess after snack	2.57	1.02	**0.72**	0.11	0.10	0.12	0.56
31. Helps put things away after shopping	2.23	1.16	**0.72**	0.02	0.19	0.02	0.56
18. Picks up dirty clothes after changing	2.81	1.05	**0.71**	0.14	0.17	0.10	0.56
4. Has regular chores	2.27	1.05	**0.68**	− 0.04	0.30	0.14	0.57
5. Straightens bedroom daily	1.98	1.01	**0.67**	0.04	0.03	− 0.08	0.45
19. Washes hands before mealtime	3.29	0.85	**0.44**	0.28	0.06	0.14	0.30
28. Picks up toys and puts them away	2.68	0.89	**0.39**	0.13	0.21	0.09	0.22
11. Does the same things before bed	3.62	0.62	0.29	**0.69**	0.25	− 0.03	0.62
21. Goes to bed at the same time (weekdays)	3.65	0.66	0.08	**0.67**	− 0.07	0.04	0.46
29. Eats breakfast at the same time/place	3.71	0.57	0.04	**0.61**	0.00	0.10	0.39
13. Wakes up at the same time (weekdays)	3.75	0.53	0.02	**0.61**	0.00	0.29	0.45
7. Hugs/kisses parents before bed	3.71	0.65	0.11	**0.59**	0.20	− 0.05	0.41
16. Eats dinner at the same time	3.62	0.55	0.10	**0.53**	0.08	0.14	0.31
1. Has a set routine for getting ready (morning)	3.72	0.60	0.15	**0.51**	0.26	− 0.02	0.35
17. Brushes teeth before bed	3.59	0.69	− 0.03	**0.51**	0.24	0.07	0.32
6. Eats meals with family at the table	3.80	0.54	0.05	**0.40**	0.16	0.12	0.20
38. Completes homework	3.63	0.77	0.01	0.08	**0.71**	0.23	0.57
37. Is supervised by an adult	3.46	0.80	0.00	0.00	**0.64**	0.17	0.44
35. Shows parent school homework	3.29	0.90	0.26	0.16	**0.63**	− 0.06	0.49
36. Begins homework at the same time/place	3.23	0.87	0.25	0.10	**0.61**	0.12	0.46
39. Studies for tests	3.32	0.93	0.30	0.20	**0.57**	− 0.04	0.45
26. Helps decide/prepare for family fun/events	2.88	0.89	0.45	0.20	**0.48** ^a^	− 0.12	0.49
14. Must finish household responsibilities	3.53	0.65	0.14	0.24	**0.45**	0.33	0.39
9. Talks with parent each day	3.55	0.66	0.28	0.34	**0.44**	− 0.18	0.43
3. Talks about their day	2.99	0.86	0.40	0.27	**0.41** ^a^	− 0.12	0.41
34. Takes part in “family time” each week	3.29	0.85	0.27	0.23	**0.38**	0.14	0.29
25. Is disciplined for misbehavior	3.01	1.00	0.02	0.09	0.00	**0.80**	0.65
27. Receives smaller punishment	2.89	1.10	0.02	− 0.01	0.06	**0.78**	0.61
12. Has household rules	3.43	0.74	0.25	0.18	0.14	**0.45**	0.32
23. Has time limits on fun activities	2.80	0.98	0.43	0.03	0.23	**0.42** ^a^	0.41
15. Receives rewards for good behavior	2.70	0.99	0.03	0.16	0.06	**0.41**	0.20
Eigenvalues			4.52	3.61	3.51	2.29	
% of Variance			14.14	11.27	10.98	7.15	

*Note*. Values in bold indicate the inclusion of the items on the respective components. ^a^ Cases of cross loadings. CRQ-PT = Child Routines Questionnaire-Portuguese Version

Table 2 shows the final solution of 32 items, that consisted of four components that explained 43.53% of the total variance. Considering the final solution of 32 items, three (items 3, 23 and 26) showed cross loadings into two factors with very similar values. First, item 23 was allocated to the fourth component, according to the original version of the CRQ. However, none of the other two variables (i.e., items 3 and 26) loaded on the respective component of the original structure of CRQ. Thus, both items were assigned to the third component based on their theoretical content. Due to the final solution obtained in the EFA, the Homework Routines subscale had to be relabelled (i.e., Homework Routines and Family Interaction), since new items were now associated with this component. As such, in the Portuguese version of the CRQ, the first component, identified as Household Responsibilities, is composed of eight items and explains 14.14% of the total variance. The second component, related to Daily Living Routines, includes nine items and accounts for 11.27% of the total variance. The third component, named Homework Routines and Family Interaction, accounts for 10.98% of the total variance and is composed of 10 items. Finally, a fourth component, identified as Discipline Routines explains 7.15% of the total variance and contains five items.

### Internal Consistency

To analyze the internal consistency of the exploratory four-factor solution, Cronbach’s alpha reliability coefficients were calculated (Table 3). A value of 0.89 was obtained for the Total scale and for the four subscales reliability coefficients were: 0.84 for Household Responsibilities, 0.78 for Daily Living Routines, 0.82 for Homework Routines and Family Interaction, and 0.63 for Discipline Routines. According to the results presented in Table 3, correlations between the four subscales are significant (*p* < .001) and positive, ranging from 0.27 to 0.57. The correlations between each subscale and the Total CRQ-PT score were also positive and significant, ranging from 0.57 (Discipline) to 0.85 (Homework/Family Interaction).

**Table 3:** Inter-correlations, means, standard-deviations and Cronbach’s alpha: CRQ-PT subscales and Total score

	1	2	3	4	*M* (*SD*)	*Cronbach*’ *α*
1. Household Responsibilities					2.65 (0.59)	0.84
2. Daily Living	0.33**				3.64 (0.40)	0.78
3. Homework and Family Interaction	0.57**	0.49**			3.39 (0.47)	0.82
4. Discipline	0.32**	0.27**	0.31**		2.96 (0.48)	0.63
5. Total CRQ-PT	0.82**	0.66**	0.85**	0.57**	3.16 (0.37)	0.89

*Note.* CRQ-PT = Child Routines Questionnaire-Portuguese Version

**p < .01

### Confirmatory Factor Analysis

The four-factor structure obtained with EFA was submitted to CFA using another sample (Study 2). The ML estimation method was used to test the suitability of the CRQ-PT four-factor structure for the Portuguese data. Prior to parameters specification, three conditions were established: (a) each variable loads on just one factor, (b) the factors are inter-correlated, and (c) only correlations between error measurements associated with items of the same factor can be estimated.

**Fig. 1:**
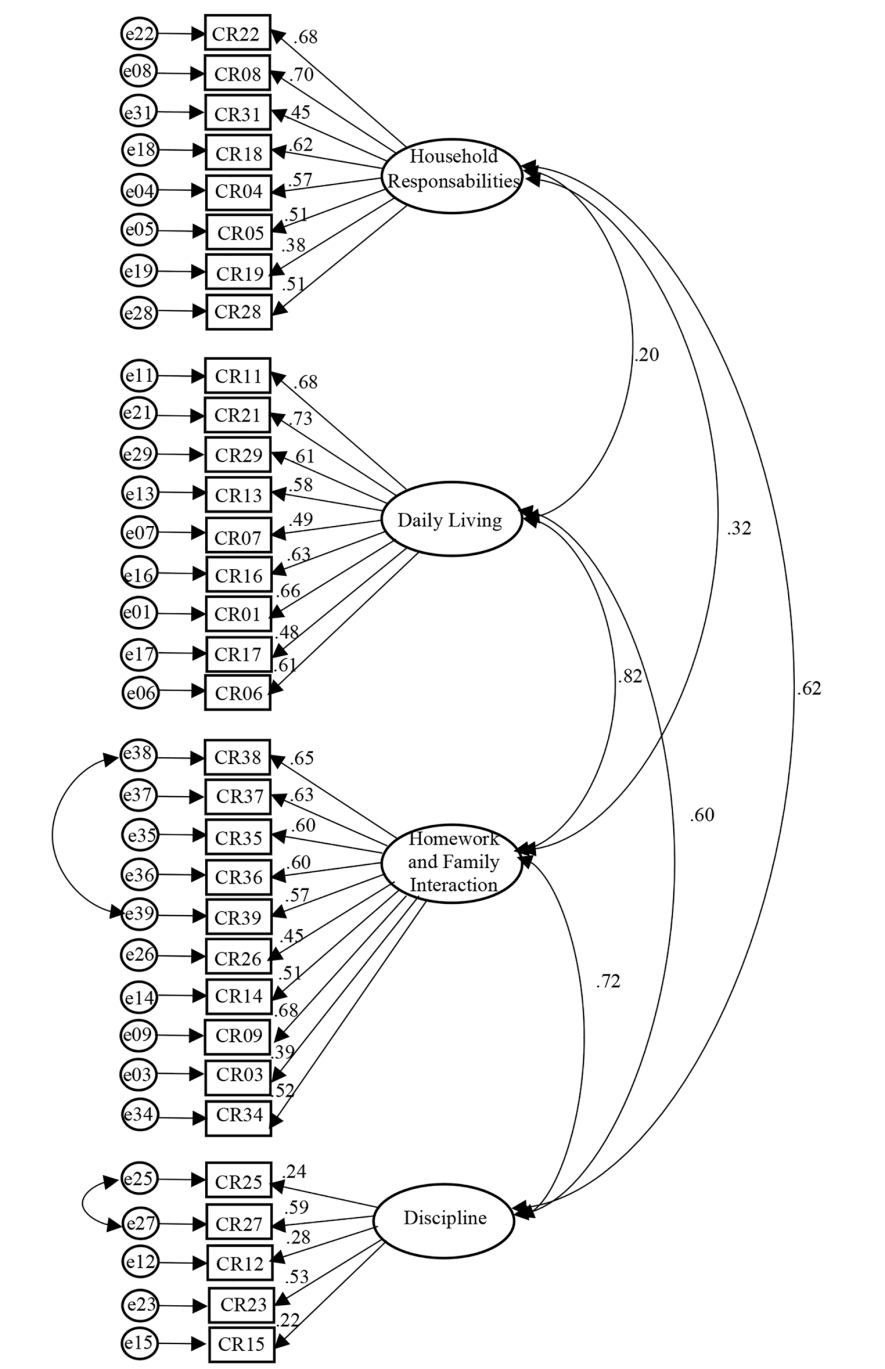
Confirmatory factor analysis of the CRQ-PT: Four-factor model, standardized regression weights and correlations across factors

The CFA revealed that the model did not sufficiently fit to the data, *χ*^*2*^(458) = 930.53, *p* < .001 *χ*^*2*^*/df* = 2.03, CFI = 0.80, GFI = 0.81 and RMSEA = 0.064 [90% CI 0.06-0.07], and the modification indices provided indications for a model improvement. Therefore, after adding covariances between two error pairs involving items with similar content (see Fig. 1; Homework and Family Interaction: items 38 and 39, and Discipline: items 25 and 27), the results indicated a more adequate model fit, *χ*^*2*^(456) = 822.25, *p* < .001.; *χ*^*2*^*/df* = 1.80; CFI = 0.84; GFI = 0.83; RMSEA = 0.056 [90% CI 0.05-0.06]. Furthermore, regarding the local adjustment of the items, all of them presented significant non-standardized regression weights (*p* > .05) and critical values below 1.96. However, three items from the Discipline subscale (i.e., items 15, 25 and 27) showed standardized estimates lower than 0.40 (i.e., between 0.22 and 0.28) and *R*^*2*^ values inferior to 0.10 (i.e., between 0.05 and 0.08).

Additionally, CFA were also run for concurrent model structures (i.e., one, two and three factors), following Byrne’s (2010) recommendation. In Table 4, fit indexes obtained for each of the four models are presented. The correlations between the errors of the same variables were specified when running the other three models. The results showed that the four-factor model was the structure that presents the better fit, considering the values of all the calculated indexes. Accordingly, both *χ*^*2*^ and *χ*^*2*^*/df* values progressively decrease as the models include more factors.

**Table 4:** Model fit indexes of CFA concurrent models: CRQ-PT

	*χ* ^*2*^	*df*	*p*	*χ* ^*2*^ */df*	GFI	CFI	RMSEA	*p*
Independent model	2845.15	496						
1-factor^a^	1226.81	462	< .001	2.66	0.71	0.67	0.081	< .001
2-factors^b^	940.83	461	< .001	2.04	0.80	0.80	0.064	< .001
3-factors^c^	884.13	459	< .001	1.92	0.81	0.82	0.060	< .001
4-factors^d^	822.25	456	< .001	1.80	0.83	0.84	0.056	> .05

*Note*. CRQ-PT = Child Routines Questionnaire-Portuguese Version

^a^All the items loaded into one single factor. ^b^Factor 1 = Household Responsibilities + Discipline; Factor 2 = Daily Living + Homework and Family Interaction. ^c^Factor 1 = Household Responsibilities; Factor 2 = Discipline; Factor 3 = Daily Living + Homework and Family Interaction. ^d^Factor 1 = Household Responsibilities; Factor 2 = Daily Living; Factor 3 = Homework and Family Interaction; Factor 4 = Discipline.

### Construct Validity

The correlations between CRQ-PT subscales and other variables are presented in Table 5. First, a measure of a routines-related construct (i.e., family mealtime routine) was considered. The four CRQ-PT subscales were positively and significantly related to the FRQ subscale for dinner time family routine (*r* = 0.16 to 0.37, *p* < .05). Also, the correlation between the CRQ-PT Total score and the FRQ subscale was positive and significant (*r* = 0.35, *p* < .001). Second, the associations between child routines subscales and the Total scale score and subscales from a parenting sense of competence measure were calculated. The results indicated that the CRQ-PT Discipline subscale was not significantly correlated with any of the PSOC subscales, *p* > .05. For the other three CRQ-PT subscales, correlation coefficients ranged from 0.16 to 0.38 for the Satisfaction subscale and from 0.13 to 0.32 for the Efficacy subscale. Finally, the association between the CRQ-PT Total score and the PSOC scores was positive and significant, ranging from 0.25 (Efficacy subscale) to 0.31 (Satisfaction subscale).

**Table 5:** Correlations between CRQ-PT, FRQ and PSOC scores

	FRQ	PSOC	
CRQ-PT Scores	Mealtime Routine	Satisfaction	Efficacy
1. Household Responsibilities	0.16*	0.16**	0.13*
2. Daily Living	0.37***	0.35***	0.23***
3. Homework and Family Interaction	0.34***	0.38***	0.32***
4. Discipline	0.19*	0.07	0.09
5. Total CRQ-PT	0.35***	0.31***	0.25***

*Note*. CRQ-PT = Child Routines Questionnaire-Portuguese Version. FRQ = Family Rituals Questionnaire. PSOC = Parenting Sense of Competence Scale

*p < .05; **p < .01; ***p < .001

## Discussion

Despite the recognized importance of routines in the development of young children (e.g., Brody & Flor 1997; Ren & Xu, 2019), in Portugal no instruments were available to assess routines of school age children. Thus, the goal of this study was to translate, adapt and validate the CRQ (Sytsma et al., 2001) for Portuguese school-age children.

An EFA was run to explore the factor structure of the CRQ-PT. Out of the 39 original items, four were previously excluded: three items of the CRQ Defensive Responding scale (Sytsma et al., 2001) and item 33 (“praying before meals”). The removal of items related to religious practices was consistent with the validation procedures of the CRQ-IS (Halldórsdóttir & Óskarsdóttir, 2009) and of the Portuguese version of the CRQ for preschool children (CRQ:P; Cunha et al., 2021). In fact, Coutinho (2019) found that in recent decades there has been a decrease in religious practices in Portuguese families, pointing to increased family education as one of the reasons. Thus, the remaining 35 items were subject to EFA and a four-component solution with 32 items was found to better represent the data, after removing three other items (2, 24 and 32) with low loadings. This solution was consistent with the four-factor factor structure of the original version of the CRQ and the CRQ-IS. For the first subscale, corresponding to Household Responsibilities (eight items), two items of the original version of the CRQ (24 and 33, which were previously removed) are missing and item 31 (“helps put things away after shopping”) was added to this subscale. This inclusion is theoretically justifiable, given that helping to unpack groceries is compatible with the content of other items of this subscale, such as “clean up the room” or “clean up the toys”. It is also consistent with a CFA of the original CRQ, which demonstrated stronger loading of item 31 on the Household Responsibilities component than on the Discipline Routines component (Jordan et al., 2006) and with EFA from the CRQ-IS where this item also loaded on the Household Responsibilities component (Halldórsdóttir & Óskarsdóttir, 2009). Furthermore, helping to unpack reflects a routine more related to household responsibilities than to discipline (as it is assigned in the original version). The second subscale, related to Daily Living Routines, includes nine items, related to child’s activities of daily living (e.g., morning routine, bedtime routine, meals). The Portuguese version included all the items from the original CRQ version, with the exception of items 3 (“takes turns with family members talking about their day”) and 9 (“spends special time talking with parent”), which were both included in the newly labelled Homework Routines and Family Interaction subscale. The third subscale, originally designated Homework Routines, is composed of 10 items in the Portuguese version, and was renamed as Homework Routines and Family Interaction, since it includes a combination of items associated with homework routines and communication, sharing daily events and family life, in line with the CRQ-IS, which has a subscale entitled Family Interaction. As such, this subscale includes all the five items from the original Homework Routines, plus five other items. Two of them (3 and 9) were switched from the original Daily Living Routines subscale, and as they refer to a routine of talking to parents and with other family members about the day, the items are consistent with communication, sharing events and a connection between parents and children, that is, family interaction. Also, item 14 was included in this subscale, due to its emphasis on completing the homework before playtime. Finally, items 26 and 34, related to family time together but originally included in Discipline Routines, were assigned to this subscale given their theoretical content. According to the literature, leisure activities at school age can be characterized as positive experiences that promote family interaction, as they are carried out together by the family members (Hodge et al., 2015). Finally, the fourth and smallest subscale, identified as Discipline Routines, is composed of five of the original items which refer to rules, discipline methods and structured family tasks. Of the remaining six items included in the original version, two were eliminated due to their low factor loadings (items 2 and 32), three are now included in the Homework Routines and Family Interaction subscale (items 14, 26 and 34), and item 31 is allocated to the Household Responsibilities subscale. In contrast with our findings, a CFA of the original school-age CRQ showed that item 34 cross loaded on both the Discipline Routines and Daily Living Routines components, rather than the Homework Routines component. This suggests that additional attention should be given to this item in future studies.

For internal consistency, the coefficient α value of 0.89 for the Total score is very close to the original version (α = 0.90; Sytsma et al., 2001) and higher than the CRQ-IS (α = 0.83; Halldórsdóttir & Óskarsdóttir, 2009). For the subscales, respectable/good to very good reliability levels were achieved, except for the Discipline Routines which remained at an undesirable level according to DeVellis (2016) guidelines. This weaker internal consistency for the Discipline Routines items was expected based on its reduction to less than half the original number of items, as compared to the original version, and the identified issues with three items during CFA. Furthermore, a relatively low internal consistency in the Discipline Routines subscale was also found in the CRQ:P (Cunhar et al., 2021). Inter-correlations between the four CRQ-PT subscales were positive weak to moderate, and moderate to high positive correlations were found between each subscale and the Total score, which highlights that each subscale assesses an independent domain of routines of school-aged children.

A CFA was then undertaken to assess the model fit of the CRQ-PT to the sample from Study 2 based on the factor structure obtained in Study 1. The first CFA revealed that the model did not sufficiently fit to the data. Based on modification indices, covariances were added to improve the model fit, between errors of items 38 and 39 from the Homework and Family Interaction subscale according to their focus on school related tasks, such as “completes homework” and “studies for tests”. The same procedure was used for items 25 and 27 from the Discipline subscale, based on their theoretical content, which have similar and comparable terminologies, such as “is disciplined” and “receives smaller punishments”. The results indicated that most of the global adjustment quality indices had acceptable values. Some concerns probably related to the item content were identified for items 15 (“Receives rewards for good behavior”), 25 (“Is disciplined for misbehavior”) and 27 (“Receives smaller punishment”) of the Discipline subscale. Thus these items might be considered potentially problematic in terms of their construct validity, and may have affected the global model fit.

As expected, the significant association between child routines and a measure of family routines (family dinner time routine) suggests evidence of construct validity. Despite the lower level of reliability achieved in the present study for the FRQ, for which the reduced number of items may have contributed, it is commonly used with Cronbach alpha values around 0.70 (e.g., Friend et al., 2015; Jones et al., 2014). Also, it was the only measure available in Portuguese to assess family routines. As for the Portuguese translation of the CRQ:P (Cunha et al., 2021), positive weak to moderate correlations were obtained between the four CRQ:P subscales and the Total score with the FRQ subscale for dinner time family routines. This lower association, as compared to studies using other family routines tools (e.g., Sytsma et al., 2001) may be explained due to the specific item content of the FRQ subscale (dinner time). In fact, in line with Sytsma et al. (2001), the highest correlation that was found referred to the Daily Living subscale, which includes items related to meals. On the other hand, the association between the Homework and Family Interaction subscale and the Dinner Time subscale of the FRQ may be justified by the fact that dinnertime is considered a time for family interaction, which promotes communication and information sharing and that enables the family members to show concern for each other’s daily lives, thus increasing interpersonal involvement (Fiese et al., 2006).

As expected and consistent with the literature that suggests that higher levels of child routines are associated with higher levels of efficacy and satisfaction derived from the parenting role (e.g., Jordan, 2003), the results showed significant correlations between the CRQ-PT and the PSOC scores. The exception was the Discipline subscale, with non-significant correlations with any of the PSOC subscales, which deserves further attention in future studies, due to its reduced size and previously identified problematic functioning in CFA. This subscale may be less associated with regular family routines and more with the sporadic use of discipline strategies to control or reduce child misbehavior. And these interactions between parents and children may not contribute to increase parental sense of self-efficacy and satisfaction. Contrastingly, both Daily Living and Homework and Family Interaction subscales showed moderate correlations with the PSOC Satisfaction and Efficacy subscales, suggesting that the more children engage in routine practices, the greater the sense of competence of parents and vice-versa. Other studies have shown that the frequent practice of routines allows parents to regulate children’s behavior, which provides an increase in parental feelings of competence (e.g., Migliorini et al., 2016), and that positive routines are associated with positive parental competence (Fiese et al., 2002; Spagnola & Fiese, 2007).

This study has some limitations which should be addressed in future studies. First, a convenience sample was used, which raises questions about its representativeness. Differences between children’s age groups in the two studies (EFA and CFA) should also be noted, since the practice of routines decreases as children get older (Cunha et al., 2021; Malenfant, 2006). Despite being more the rule than the exception, the participants were mostly mothers. In fact, this is a common situation in different countries (e.g., Henderson et al., 2011; Stoppelbein et al., 2016) and similar to studies conducted in the US with the CRQ. Thus, it is recommended the use of larger samples and a greater participation of fathers in future research. It would be important to collect data from both parents to assess the informant agreement, as they are the reference informants regarding child routines. The poorer adjustment and lower internal consistency obtained with the Discipline Routines subscale emphasizes the need of further attention in future studies. The addition of more items to the CRQ-PT that could describe more relevant daily parenting discipline rules and practices may contribute to higher internal consistency and robustness of Discipline Routines construct.

The development of further studies of reliability (e.g., temporal stability) and validity evidence with other measures (e.g., behavior problems, social skills, academic achievement) or special groups (e.g., children with ADHD, ASD) that may reinforce the psychometric properties of the CRQ-PT is also suggested. Some items from the Discipline Routines subscale showed relatively poor functioning in the CFA; as such, in the future it would be relevant to clarify their suitability in the assessment of the constructs measured by the CRQ-PT and its subscales. Also, longitudinal studies could be undertaken to understand the long-term impact of positive and sustained routines in child and adolescent development, as well as in adult adjustment. Finally, and considering the recent pandemic context and all the changes that have occurred in the daily lives of families (e.g., lockdown, telework, e-learning), it might be equally relevant to develop studies that assess the impact of the COVID-19 pandemic in the routines of families with school-age children.

The present study offers relevant contributions to the assessment of the routines of school-age children in the Portuguese context. The factor structure of the CRQ-PT was examined through exploratory and confirmatory studies and overall, the results provided good reliability indices, which reinforces the psychometric properties of the measure. It is now available as an instrument to be used in clinical practice, but also in research with children of school age (6 to 12 years old). Furthermore, having a school-age version of the CRQ allows a continuity in the assessment of child routines, since the preschool version (CRQ:P; Cunha et al., 2021) recently became available in Portugal. Finally, this study contributed to an additional understanding of the relationship between the perception of parental competence and routines practices of school-age children, an area where studies are scarce.

## References

[CR1] Cunha, A. I., Major, S., Alves, M. P., & Coroado, M. (2021). Assessing preschool child routines in the family: A preliminary study of the Portuguese version of the Child Routines Questionnaire - Preschool. *Journal of**Research in Childhood Education*, *36*(2), 310-326. https://doi.org/10.1080/02568543.2021.1955053

[CR2] Jordan, S. S. (2003). *Further validation of the Child Routines Inventory (CRI): Relationship to parenting practices, maternal distress and child externalizing behavior* [Doctoral dissertation, Louisiana State University]. LSU Digital Commons. https://digitalcommons.lsu.edu/gradschool_dissertations/3308

[CR3] Sytsma, S. E., Kelley, M. L., & Wymer, J. H. (2001). Development and initial validation of the child routines inventory. *Journal of Psychopathology and Behavioral Assessment*, *23*(4), 241-251. https://doi.org/10.1023/A:1012727419873 10.1007/s10862-022-10007-7 PMC9734787 36531436

[CR4] Jordan, S. S., Arnau, R. C., Stoppelbein, L., Greening, L., & Henderson, J. (2006). *Confirmatory factor analysis of the Child Routines Questionnaire*. A poster presentation at the 40th Annual Meeting of the Association for Behavioral and Cognitive Therapies, Chicago, IL.

[CR5] Bennett DA (2001). How can i deal with missing data in my study?. Australian and New Zealand Journal of Public Health.

[CR6] Bridley A, Jordan SS (2012). Child routines moderate daily hassles and children’s psychological adjustment. Children’s Health Care.

[CR7] Brody GH, Flor DL (1997). Maternal psychological functioning, family processes, and child adjustment in rural, single-parent, african american families. Developmental Psychology.

[CR8] Bryman, A., & Cramer, D. (2004). *Quantitative data analysis with SPSS 12 and 13: a guide for social scientists*. Routledge.

[CR9] Byrne, B. M. (2010). *Structural equation modelling with AMOS: basic concepts, applications, and programming* (2nd ed.). Routledge.

[CR10] Cattell RB (1966). The Scree test for the number of factors. Multivariate Behavioral Research.

[CR11] Costello AB, Osborne JW (2005). Best practices in exploratory factor analysis: four recommendations for getting the most from your analysis. Practical Assessment Research and Evaluation.

[CR12] Coutinho JP (2019). Religiosidade em Portugal: Caracterização, comparação e evolução [Religiosity in Portugal: characterization, comparison and evolution]. Religião & Sociedade.

[CR13] Crespo C, Davide I, Costa M, Fletcher G (2008). Family rituals in married couples: links with attachment, relationship quality, and closeness. Personal Relationships.

[CR14] DeVellis, R. F. (2016). *Scale development: theory and applications* (4th ed.). Sage.

[CR15] Evans DW, Leckman JF, Carter A, Reznick JS, Henshaw D, King RA, Pauls D (1997). Ritual, habit, and perfectionism: the prevalence and development of compulsive-like behavior in normal young children. Child Development.

[CR16] Evans J, Rodger S (2008). Mealtimes and bedtimes: Windows to family routines and rituals. Journal of Occupational Science.

[CR17] Eyberg SM, Ross AW (1978). Assessment of child behavior problems: the validation of a new inventory. Journal of Clinical Child Psychology.

[CR18] Ferretti LK, Bub KL (2017). Family routines and school readiness during the transition to kindergarten. Early Education and Development.

[CR19] Fiese, B. H., Foley, K. P., & Spagnola, M. (2006). Routine and ritual elements in family mealtimes: Contexts for child well- being and family identity. In R. W. Larson, A. R. Wiley, & K. R. Branscomb (Eds.), *Family mealtime as a context of development and socialization* (pp. 67–89). Wiley. 10.1002/cad.155 10.1002/cd.156 16646500

[CR20] Fiese BH, Kline CA (1993). Development of the family ritual questionnaire: initial reliability and validation studies. Journal of Family Psychology.

[CR21] Fiese BH, Tomcho TJ, Douglas M, Josephs K, Poltrock S, Baker T (2002). A review of 50 years of research on naturally occurring family routines and rituals: cause for celebration?. Journal of Family Psychology.

[CR22] Friend S, Fulkerson JA, Neumark-Sztainer D, Garwick A, Flattum CF, Draxten M (2015). Comparing childhood meal frequency to current meal frequency, routines and expectations among parents. Journal of Family Psychology.

[CR23] Gjersing L, Caplehorn JR, Clausen T (2010). Cross-cultural adaptation of research instruments: Language, setting, time and statistical considerations. BMC Medical Research Methodology.

[CR24] Greening L, Stoppelbein L, Konishi C, Jordan SS, Moll G (2007). Child routines and youths’ adherence to treatment for type 1 diabetes. Journal of Pediatric Psychology.

[CR25] Halldórsdóttir, B. K., & Óskarsdóttir, H. (2009). *Próffræðilegir eiginleikar íslenskrar útgáfu Child Routines Questionnaire* [*Psychometric properties of the Icelandic version of the Child Routine Questionnaire*] [Bachelor thesis, University of Island]. Skemman. http://hdl.handle.net/1946/2857

[CR26] Henderson JA, Barry TD, Bader SH, Jordan SS (2011). The relation among sleep, routines, and externalizing behavior in children with an autism spectrum disorder. Research in Autism Spectrum Disorders.

[CR27] Hodge C, Bocarro JN, Henderson KA, Zabriskie R, Parcel TL, Kanters MA (2015). Family leisure: an integrative review of research from select journals. Journal of Leisure Research.

[CR28] Howe GW (2002). Integrating family routines and rituals with other family research paradigms: comment on the special section. Journal of Family Psychology.

[CR29] Jackson DL, Gillaspy JA, Purc-Stephenson R (2009). Reporting practices in confirmatory factor analysis: an overview and some recommendations. Psychological Methods.

[CR30] Jensen EW, James SA, Boyce WT, Hartnett SA (1983). The Family Routines Inventory: Development and validation. Social Science & Medicine.

[CR31] Johnston C, Mash EJ (1989). A measure of parenting satisfaction and efficacy. Journal of Clinical Child Psychology.

[CR32] Jones BL, Fiese BH, STRONG Kids Team (2014). Parent routines, child routines, and family demographics associated with obesity in parents and preschool-aged children. Frontiers in Psychology.

[CR33] Kitsaras G, Goodwin M, Allan J, Kelly MP, Pretty IA (2018). Bedtime routines child wellbeing & development. Bmc Public Health.

[CR34] Malenfant, N. (2006). *Routines and transitions: a guide for early childhood professionals*. Readleaf Press.

[CR35] McCloy L, White S, Bunting KL, Forwell S (2016). Photo-elicitation interviewing to capture children’s perspectives on family routines. Journal of Occupational Science.

[CR36] McRae E, Stoppelbein L, O’Kelley S, Fite P, Smith S (2020). Comorbid internalizing and externalizing symptoms among children with ADHD: the influence of parental distress, parenting practices, and child routines. Child Psychiatry and Human Development.

[CR37] Meyer, K. (2008). *Development and Initial validation of the adolescent routines questionnaire: Parent and self-report* [Doctoral dissertation, Louisiana State University]. LSU Digital Commons. https://digitalcommons.lsu.edu/gradschool_dissertations/4052

[CR38] Migliorini L, Cardinali P, Rania N (2011). La cotidianidad de lo familiar y las habilidades de los niños. Psicoperspectivas.

[CR39] Migliorini L, Rania N, Tassara T, Cardinali P (2016). Family routine behaviors and meaningful rituals: a comparison between italian and migrant couples. Social Behavior and Personality.

[CR40] Mindell JA, Williamson AA (2018). Benefits of a bedtime routine in young children: Sleep, development, and beyond. Sleep Medicine Reviews.

[CR41] Muñiz EI, Silver EJ, Stein REK (2014). Family routines and social-emotional school readiness among preschool-age children. Journal of Developmental and Behavioral Pediatrics.

[CR42] Nymoen, K. (2014). *The effects of parent training on child routines* [Master’s thesis, University of Island]. Skemman. http://hdl.handle.net/1946/17644

[CR43] Pennick, M. R. (2013). *Understanding the relation between routines and problem behaviors in children with clinical diagnoses* (UMI Number: 3561304) [Doctoral dissertation, University of Alabama at Birmingham]. ProQuest.

[CR44] Rania N, Migliorini L, Rebora S (2018). Parental competence in Italy: a comparison between italian and immigrant parents. Marriage and Family Review.

[CR45] Ren H, He X, Bian X, Shang X, Liu J (2021). The protective roles of exercise and maintenance of daily living routines for chinese adolescents during the COVID-19 quarantine period. Journal of Adolescent Health.

[CR46] Ren L, Fan J (2019). Chinese preschoolers’ daily routine and its associations with parent-child relationships and child self-regulation. International Journal of Behavioral Development.

[CR47] Ren L, Xu W (2019). Coparenting and chinese preschoolers’ social-emotional development: child routines as a mediator. Children and Youth Services Review.

[CR48] Seabra-Santos MJ, Major S, Pimentel M, Gaspar MF, Antunes N, Roque V (2015). Escala de sentido de competência parental (PSOC): Estudos psicométricos [*Parenting sense of competence scale (PSOC): psychometric studies*]. Avaliação Psicológica.

[CR49] Shrestha N (2021). Factor analysis as a tool for survey analysis. American Journal of Applied Mathematics and Statistics.

[CR50] Spagnola M, Fiese BH (2007). Family routines and rituals: a context for development in the lives of young children. Infants & Young Children.

[CR51] Sprunger LW, Boyce WT, Gaines JA (1985). Family-infant congruence: routines and rhythmicity in family adaptations to a young infant. Child Development.

[CR52] Stabler, C. B. (2012). *Further validation of the Child Routines Questionnaire: Child Self Report* [Doctoral dissertation, University of Southern Mississippi]. The Aquila Digital Community. https://aquila.usm.edu/dissertations/591

[CR53] Stoppelbein L, Biasini F, Pennick M, Greening L (2016). Predicting internalizing and externalizing symptoms among children diagnosed with an autism spectrum disorder: the role of routines. Journal of Child and Family Studies.

[CR54] Tabachnick, B. G., & Fidell, L. S. (2007). *Using multivariate statistics* (5th ed.). Allyn and Bacon.

[CR55] Urcuioli PJ (2005). Behavioral and associative effects of differential outcomes in discrimination learning. Learning and Behavior.

[CR56] Weisner TS (2002). Ecocultural understanding of children’s developmental pathways. Human Development.

[CR57] Wittig, M. M. (2005). *Development and validation of Child Routines Questionnaire: Preschool* [Doctoral dissertation, Louisiana State University]. LSU Digital Commons. https://digitalcommons.lsu.edu/gradschool_dissertations/1152

